# Anti-gonadotrophin releasing hormone antibodies inhibit the growth of MCF7 human breast cancer xenografts

**DOI:** 10.1038/sj.bjc.6690362

**Published:** 1999-05-01

**Authors:** E Jacobs, S A Watson, D Michaeli, I O Ellis, J F R Robertson

**Affiliations:** Professorial Unit of Surgery, City Hospital, Nottingham NG5 1PB, UK; Cancer Studies Unit, Department of Surgery, D-floor, University Hospital, Nottingham NG7 2UH, UK; Aphton Corporation, Woodland, CA 95776, USA

**Keywords:** breast cancer, xenograft, GnRH, immunotherapy

## Abstract

The human breast cancer cell line (MCF7) was established as xenografts in intact female nude mice. Xenografts did not require oestrogen supplementation for growth, although oestrogen supplementation caused more rapid tumour growth. GnRH Pharmaccine is an immunogen composed of gonadotrophin releasing hormone (GnRH) linked to diphtheria toxoid. Anti-GnRH antibodies purified from the serum of rabbits immunized with GnRH Pharmaccine, were used to passively immunize nude mice. In mice treated with anti-GnRH antibodies, xenograft growth was significantly inhibited relative to controls (median times of 71 and 29 days respectively taken for tumours to attain a predetermined cross-sectional area of 200 mm^2^, *P* < 0.001). The inhibition of tumour growth achieved by anti-GnRH antibodies was not significantly different from that produced by the anti-oestrogen, tamoxifen (59 days). Ovarian/uterine weights were reduced by 61% (*P* < 0.001) in anti-GnRH antibody-treated animals compared with controls. Histologically there was underdevelopment and atrophy of the reproductive organs. Serum levels of both oestrogen and luteinizing hormone were reduced by treatment with anti-GnRH antibodies (to 24.9% and 53% respectively of levels in controls, both *P* = 0.04). It is postulated that one of the mechanisms by which anti-GnRH antibody treatment inhibits tumour growth is indirectly, by reducing serum oestrogen levels. © 1999 Cancer Research Campaign


*British Journal of Cancer* (1999) **80**(3/4), 352–359
© 1999 Cancer Research Campaign
Article no. bjoc.1998.0362

### Anti-gonadotrophin releasing hormone antibodies inhibit the growth of MCF7 human breast cancer xenografts

**E Jacobs[2], SA Watson[2], D Michaeli[3], IO Ellis[1] and JFR Robertson[1]**

[1]Professorial Unit of Surgery, City Hospital, Nottingham NG5 1PB, UK; [2]Cancer Studies Unit, Department of Surgery, D Floor, West Block, University Hospital, Nottingham NG7 2UH, UK; [3]Aphton Corporation, Woodland, CA 95776, USA

**Summary** The human breast cancer cell line (MCF7) was established as xenografts in intact female nude mice. Xenografts did not require oestrogen supplementation for growth, although oestrogen supplementation caused more rapid tumour growth. GnRH Pharmaccine is an immunogen composed of gonadotrophin releasing hormone (GnRH) linked to diphtheria toxoid. Anti-GnRH antibodies purified from the serum of rabbits immunized with GnRH Pharmaccine, were used to passively immunize nude mice. In mice treated with anti-GnRH antibodies, xenograft growth was significantly inhibited relative to controls (median times of 71 and 29 days respectively taken for tumours to attain a predetermined cross-sectional area of 20 mm$^2$, $P < 0.001$). The inhibition of tumour growth achieved by anti-GnRH antibodies was not significantly different from that produced by the anti-oestrogen, tamoxifen (59 days). Ovarian/uterine weights were reduced by 61% ($P < 0.001$) in anti-GnRH antibody-treated animals compared with controls. Histologically there was underdevelopment and atrophy of the reproductive organs. Serum levels of both oestrogen and luteinizing hormone were reduced by treatment with anti-GnRH antibodies (to 24.9% and 53% respectively of levels in controls, both $P = 0.04$). It is postulated that one of the mechanisms by which anti-GnRH antibody treatment inhibits tumour growth is indirectly, by reducing serum oestrogen levels.

**Keywords:** breast cancer; xenograft; GnRH; immunotherapy

Oophorectomy has been the standard first line endocrine therapy in premenopausal patients with advanced breast cancer over the past century (Beatson, 1896; Lett, 1905). Initially, this was carried out either by surgery, or through the use of radiotherapy, both methods producing an irreversible post-menopausal state. More recently, GnRH agonists (e.g. goserelin) have been reported to inhibit ovarian function and reduce oestrogen ($E_2$) and progesterone to post-menopausal levels (Williams et al, 1986). The effect is reversible with ovarian function returning to normal on stopping treatment. This particular point has been used to promote gonadotrophin releasing hormone (GnRH) analogues not only for advanced breast cancer where it has been shown to be effective (Blamey et al, 1992), but also as an adjuvant breast cancer therapy and in benign gynaecological conditions. This paper reports on a new, potentially reversible, method of inhibiting ovarian function via anti-GnRH antibodies (Ab), and its effect on the in vivo growth of the hormone-sensitive breast cancer cell line, MCF7.

GnRH Pharmaccine is an immunogen consisting of the GnRH decapeptide linked through its amino terminus via a 7 amino acid spacer to diphtheria toxoid (DT). GnRH Pharmaccine has been shown to induce production of anti-GnRH Ab when injected into rabbits. This was accompanied by disruption of the normal hypothalamus/pituitary/gonadal axis resulting in the castrate state and proved to be an effective method of contraception (D Michaeli, personal communication). This alteration of hormonal status included reduced serum levels of $E_2$ in female, and testosterone in male, rabbits. The present study was established to determine whether treatment with anti-GnRH Ab would inhibit the growth of MCF7 human breast cancer xenografts in a nude mouse model and to provide information on the mechanisms involved.

*Received 7 June 1998*
*Revised 16 November 1998*
*Accepted 17 November 1998*

*Correspondence to:* E Jacobs

### MATERIALS AND METHODS

### Cell line

The human breast tumour cell line MCF7 was obtained from Dr John Nelson, Queens University, Belfast. The cell line grown in vitro in our laboratory was sensitive to $E_2$ and expressed high levels of both oestrogen (ER) and progesterone receptors (PR). Receptor levels measured in fmol mg$^{-1}$ of cytosolic protein by EIA were: ER – 128 ± 25; PR – 367 ± 13 (without $E_2$), the latter rising to 487 ± 16 (with $E_2$ supplementation) (Jacobs et al, 1996).

### Xenograft tumour

Female nude mice (Harlan-Olac, Bicester, UK) were maintained in sterile isolation (Cancer Studies Unit, University of Nottingham) and used at 6–8 weeks of age. A xenograft was established by subcutaneous (s.c.) injection of in vitro cultured MCF7 cells into the flank of mice supplemented with $E_2$. For the first experiment, the MCF7 xenograft line was maintained in passage by implanting portions of minced tumour s.c. in $E_2$-supplemented mice, with the $E_2$ pellets being removed when tumours were palpable and before treatment started. The MCF7 xenograft line used in subsequent experiments was maintained in passage by implanting portions of minced tumour s.c. in mice without $E_2$ supplementation. All animal work conformed to United Kingdom Co-ordinating Committee for Cancer Research (UKCCCR) guidelines.

**Table 1** Median time in days from start of treatment: animals bearing tumours smaller than 200 mm$^2$ cross-sectional area

| Treatment | Experiment 1 | Experiment 2 | Experiment 3 | Experiments 1–3 |
|---|---|---|---|---|
| $E_2$ | 14 | NT | NT | NA |
| 0.9% NaCl | 31.5 | 33.3 | 26.3 | 29.4 |
| Rabbit IgG | NT | NT | 35.0 | NA |
| Anti-GnRH Ab | 77.0 | 49.0 | 55.0 | 71.6 |
| Tamoxifen | 44.8[a] | 84.0 | 56.0 | 59.4 |
| Placebo | 42.0 | 35.0 | 35.0 | 37.7 |

The median times were derived using Lee–Desu statistics (Lee and Desu, 1972) for individual treatment groups (*n* = 8–10) within each experiment and (last column) in a separate analysis for all animals (*n* = 26–30) in treatment groups common to all three experiments. [a]Tumour regressed completely in one animal; NT, not tested; NA, not applicable.

### Anti-GnRH Ab treatment and control groups

1. One group of mice received passive immunization with anti-GnRH Ab that had previously been raised in rabbits; the rabbits were immunized with GnRH Pharmaccine to raise anti-GnRH Ab that was then affinity-purified from the rabbit serum. Anti-GnRH Ab was given by intraperitoneal (i.p) injection twice weekly at a dose of 0.35 mg in 0.5 ml 0.9% sodium chloride (NaCl).
2. Purified rabbit immunoglobulin (IgG) was obtained from Sigma (Poole, Dorset, UK). The dose given i.p twice weekly was 0.35 mg IgG in 0.5 ml 0.9% NaCl.
3. 0.9% NaCl (0.5 ml) was given as a control by the same schedule (i.e. twice weekly i.p.). Pellets were obtained from Innovative Research of America (Sarasota, FL, USA) and implanted s.c. in the non-tumour-bearing flank. The pellets deliver treatments (4–6) by slow release over 60 days and were replaced when necessary to maintain dosage.
4. Tamoxifen pellets (5.0 mg).
5. Corresponding placebo pellets with the same basic composition but without the active drug.
6. $E_2$ supplementation ($E_2$ pellet 0.72 mg).

### Determination of the effect of treatment on tumour growth

When xenograft tumours became palpable, the nude mice were randomized into treatment groups (*n* = 10 per group) and $E_2$ pellets removed (Experiment 1). Experiments comparing the four main treatment groups (anti-GnRH Ab, 0.9% NaCl control, Tamoxifen pellet, or placebo pellet) were performed three times.

The rationale for the treatment groups was as follows: Anti-GnRH Ab was evaluated against 0.9% NaCl, representing the baseline control (vehicle in which antibody was delivered). The therapeutic effect of anti-GnRH Ab treatment was compared with tamoxifen, a treatment known to be effective in the treatment of hormone-sensitive breast cancer given at a dose known to be effective in this xenograft model (Osborne et al, 1985). Placebo pellets were used as a control for the tamoxifen-treated group. $E_2$ supplementation was maintained in a fifth group (experiment 1) to determine the hormone sensitivity of the MCF7 xenograft. In experiment 3 an additional group was included to assess any non-specific effects of rabbit IgG on xenograft growth since anti-GnRH antibodies had been raised in rabbits.

Xenograft tumour size was measured twice weekly by an independent observer blind to which treatment the mice were receiving. The tumour size was expressed as the multiplication product of the two largest perpendicular diameters measured in mm using callipers. Animals were terminated when xenograft tumour size (cross-sectional area) exceeded 250 mm$^2$. For each of the three experiments, mean tumour size was plotted until animals started to be terminated. The time in days taken for tumour size to reach 200 mm$^2$ was noted for each animal and these data for the four treatment groups common to all three experiments (anti-

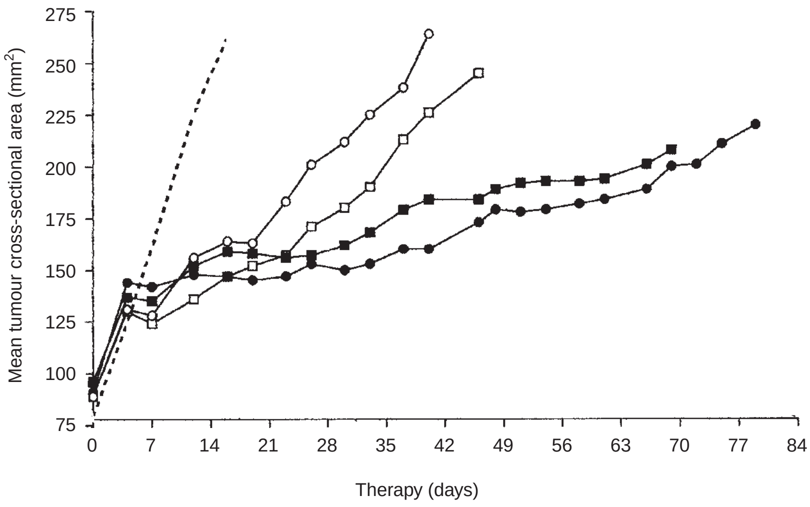

**Figure 1** Experiment 1. The mean cross-sectional area in mm$^2$ (*y* axis) of MCF7 xenografts in nude mice within each treatment group (*n* = 10) was plotted against time in days from start of therapy (*x* axis) during the period before any mice were terminated. s.e.m. are not shown for clarity. Treatments given were: 0.9% NaCl control (○), anti-GnRH antibody (●), placebo control (□), tamoxifen (■) and $E_2$ (- - -)

*© Cancer Research Campaign 1999*

**Table 2** Oestrogen and progesterone receptor expression in MCF7 xenografts

| Treatment | Experiment | ER | | PR | |
|---|---|---|---|---|---|
| | | Cells staining (%) | H-score | Cells staining( %) | H-score |
| Control (NaCl) | 1 | 70 ± 0 | 75 ± 5 | 20 ± 0 | 45 ± 0 |
| | 2 | 83 ± 8 | 153 ± 11 | < 5 | 0 |
| | 3 | 95 ± 5 | 150 ± 40 | < 5 | 0 |
| Rabbit IgG | 3 | 100 ± 0 | 190 ± 0 | < 5 | 0 |
| Anti-GnRH Ab | 1 | 90 ± 0 | 175 ± 5 | < 5 | 2.5 ± 2.5 |
| | 2 | 95 ± 5 | 205 ± 27 | < 5 | 0 |
| | 3 | 100 ± 0 | 180 ± 0 | < 5 | 0 |
| Tamoxifen | 1 | 0 | 0 | 22 ± 8 | 47 ± 18 |
| | 2 | 58 ± 34 | 95 ± 64 | 26 ± 2 | 53 ± 4 |
| | 3 | 70 ± 10 | 65 ± 15 | 5 ± 0 | 10 ± 0 |
| Placebo | 1 | 60 ± 10 | 75 ± 25 | 25 ± 10 | 52 ± 22 |
| | 2 | 87 ± 5 | 147 ± 33 | < 5 | 8 ± 11 |
| | 3 | 100 ± 0 | 175 ± 5 | < 5 | 0 |
| $E_2$ | 1 | 0 | 0 | 70 ± 0 | 120 ± 0 |

ER and PR expression was evaluated semi-quantitatively by immunocytochemical methods using receptor-specific monoclonal antibodies. The % of cells staining positive for each receptor and the H-scores derived by taking into account the intensity of staining (Goulding et al, 1995) are shown. The values given are the mean ± s.e.m. for 2–4 tumours from each treatment group in separate experiments.

GnRH Ab, 0.9% NaCl, tamoxifen pellet, or placebo pellet) were combined for analysis.

### Assessment of reproductive organs

The uteri with ovaries attached were dissected out and excess fat removed. The combined weight of ovaries and uterus was determined for individual animals in each treatment group. The organs were then fixed in formol calcium, embedded in paraffin wax, sections were cut through both ovary and uterus and stained with haematoxylin and eosin (H&E). All histological sections were examined by a consultant pathologist (IOE) who was not informed which treatment the mice had been given.

### Measurement of $E_2$ and luteinizing hormone serum concentrations

Serum was collected at 28-day intervals from start of treatment and also at termination. Samples within treatment groups were pooled to provide sufficient volumes for analysis. Serum $E_2$ levels were measured by a sensitive $E_2$ radioimmune assay (Dowsett et al, 1987). The detection limit of the assay was 3 pM per litre and the mean CV between 5 and 30 pM per litre was < 7%. Luteinizing hormone (LH) was measured using a rat LH radioimmunoassay (RIA) supplied by National Hormone and Pituitary Program (NIDDK) (anti-rat LH-S-11 antiserum lot# AFP-C697071P, rat LH-1-9 lot# AFP-10250C, rat LH-RP-3 lot# AFP-7187B).

### ER and PR measurement in tumours

ER and PR expression in xenograft tumours from each treatment group were determined semi-quantitatively by immunocytochemical methods. Paraffin sections for tumours from each treatment group were fixed and stained for ER using DAKO ID5 anti-ER monoclonal antibody (DAKO, High Wycombe, Bucks, UK) and for PR using a rat anti-PR monoclonal antibody (PR-EIA kit 4012; Abbott, Maidenhead, Berks, UK). The sections were assessed for ER and PR staining by an independent pathologist (IOE) experienced in human breast pathology. Tumour sections were scored for percentage of cells staining for ER (or PR) and for the intensity of the staining. Histochemical scores (H-scores) were calculated from these two values as previously described (Goulding et al, 1995).

### Statistical analysis

The change in size for each xenograft tumour was calculated at various time points after start of treatment. These data were analysed by analysis of variance (ANOVA) using repeated measures. The time in days for xenografts to attain 200 $mm^2$ was used as an end point and compared between treatment groups using Lee–Desu statistics which are a modification of Gehan's generalized Wilcoxon test (Lee and Desu, 1972). Tumours that did not reach 200 $mm^2$ were included in the analysis. Mann–Whitney analysis was used to compare treatment pairs at certain time points within each experiment. Mann–Whitney analysis was also used to analyse reproductive organ weights, H-scores and serum hormone levels with differences regarded as significant if $P < 0.05$.

### RESULTS

### Effect of treatment on tumour growth

The rate of tumour growth was significantly increased by $E_2$ supplementation ($P < 0.05$) (Figure 1). Rabbit anti-GnRH Ab inhibited tumour xenograft growth significantly by day 21 of treatment ($P < 0.05$ by Mann–Whitney and ANOVA) compared to 0.9% NaCl-treated control mice in all three experiments (Figures 1–3) and compared to IgG-treated control mice (experiment 3). Tumour growth was inhibited significantly in mice treated with tamoxifen compared to mice bearing placebo pellets in experiment 2 (Mann–Whitney, $P < 0.05$ at day 28, Figure 2). There was a

 © Cancer Research Campaign 1999

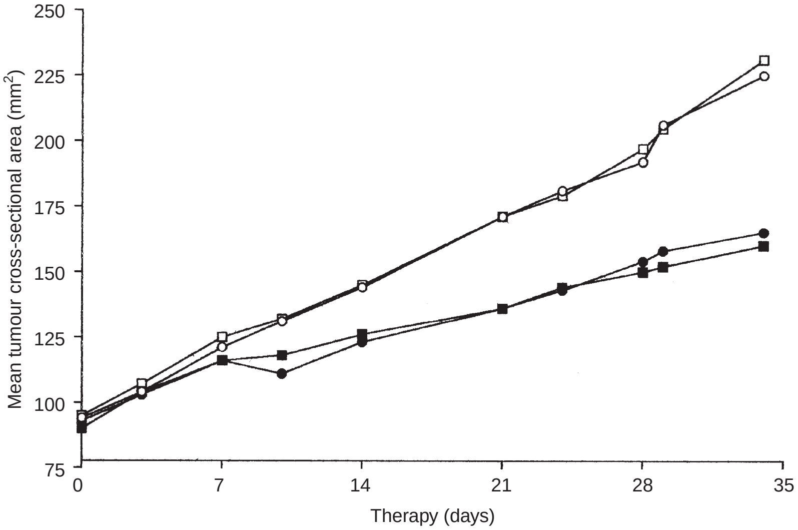

**Figure 2** Experiment 2. Coordinates and plot symbols as in Figure 1

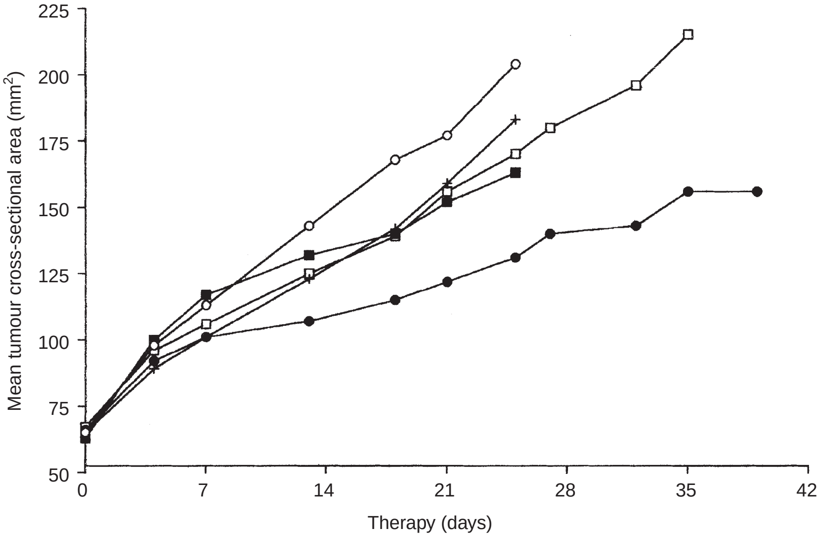

**Figure 3** Experiment 3. Coordinates and plot symbols as in Figure 1, plus treatment with rabbit IgG (+)

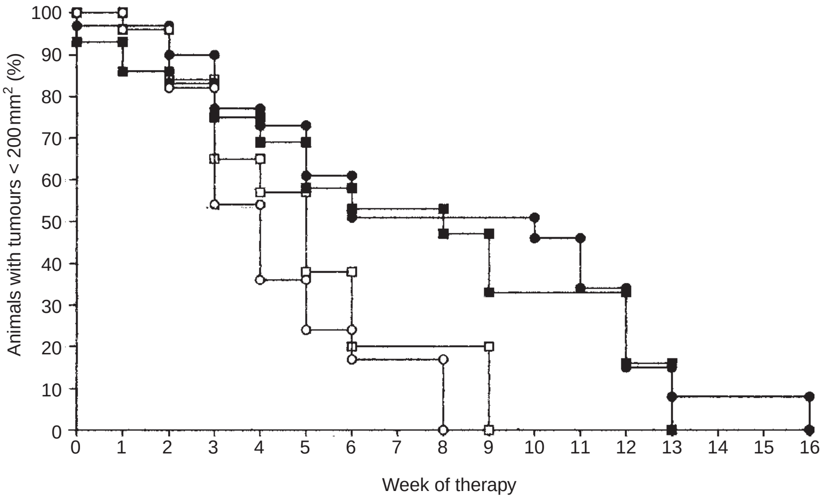

**Figure 4** The effect of treatment on tumour growth rate: the percentage of animals with tumours of cross-sectional area < 200 mm$^2$ (*y* axis) was plotted at weekly intervals (*x* axis) pooling data from all three experiments. Symbols: 0.9% NaCl control (○), anti-GnRH antibody (●), placebo control (□) and tamoxifen (■)

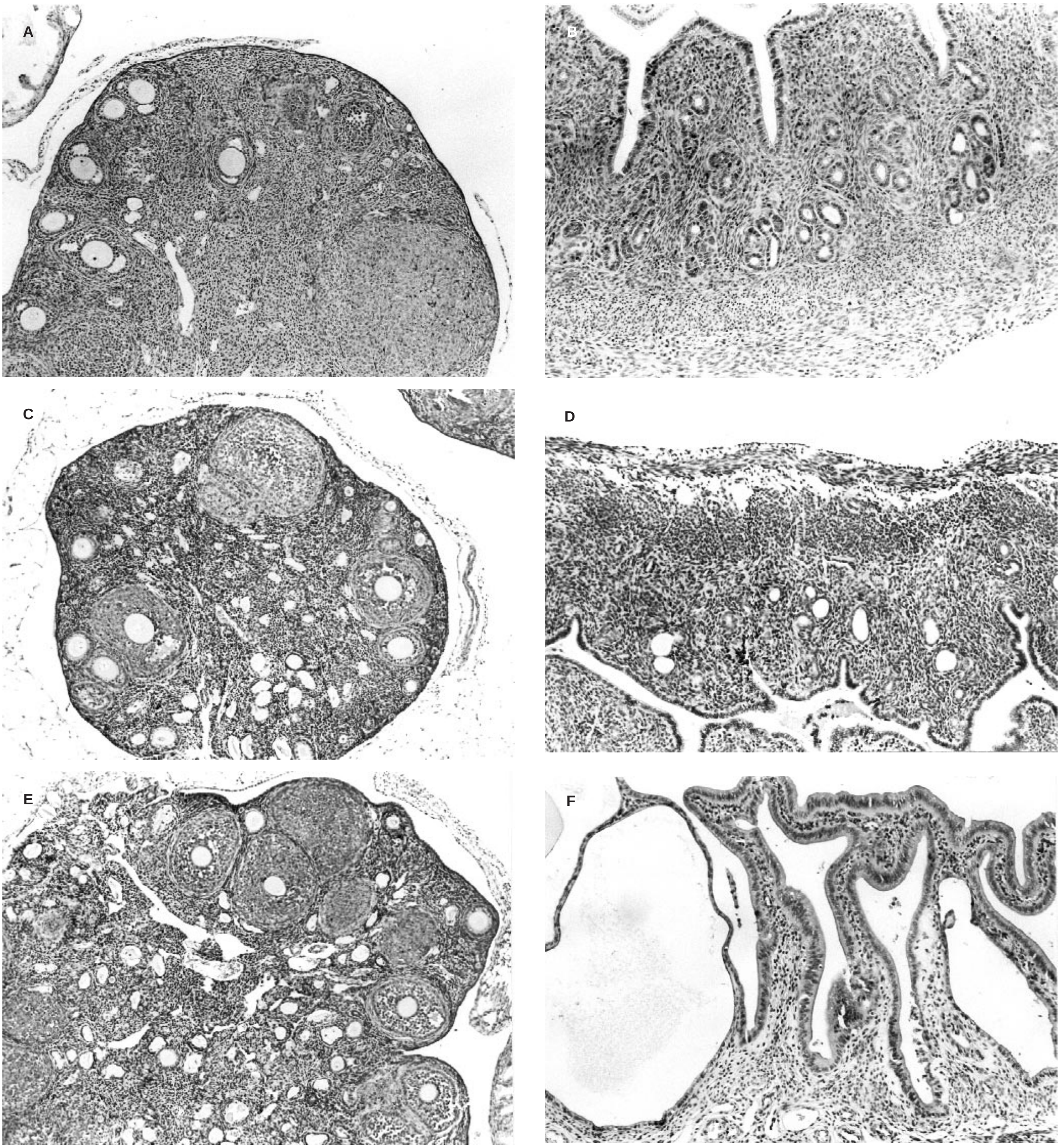

**Figure 5** Reproductive organs: sections stained with haematoxylin and eosin from control animals (**A, B**), from those treated with anti-GnRH Ab (**C, D**) and tamoxifen (**E, F**). Uteri (**B, D, F**) were photographed at double the magnification used for ovaries (**A, C, D**)

similar trend in experiment 1 (Figure 1), where one animal treated with tamoxifen showed complete regression of tumour growth. There was no difference between tamoxifen and placebo groups in experiment 3 (Figure 3) during the 3 weeks before any mice were terminated.

The effect of treatments on tumour growth over a longer time period was analysed further. Median times taken for tumours to reach 200 $mm^2$ in each of the treatment groups within individual experiments are shown (Table 1, columns 1–3). Median times (taken for tumours to reach 200 $mm^2$) were greater in treated animals than in controls (anti-GnRH Ab vs 0.9% NaCl and tamoxifen vs placebo). Data for these four groups in all three experiments were combined and are described below. The median time for tumours to reach 200 $mm^2$ in animals treated with IgG (experiment 3) was 35 days (Table 1). This was not significantly different from the results in 0.9% NaCl control groups (26.3–33.3 days). In

 *© Cancer Research Campaign 1999*

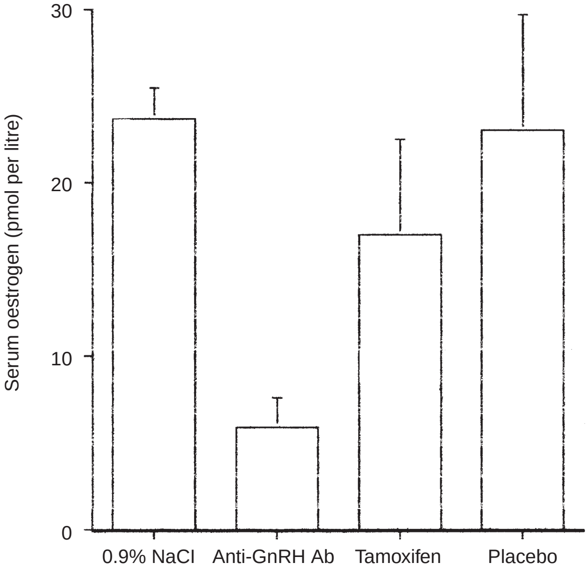

**Figure 6** E2 levels (mean ± s.e.m. for three experiments) measured in pmol per liter (*y* axis) in serum pooled for animals (*n* = 10) within each treatment group (*x* axis)

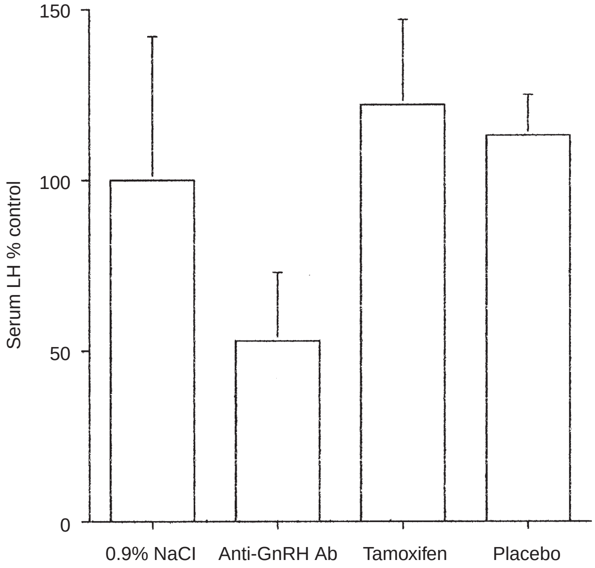

**Figure 7** LH serum levels were measured in serum pooled from animals (*n* = 10) within each treatment group and expressed as a percentage of that in controls (100%). The graph shows mean % ± s.e.m. for three experiments (*y* axis) for four treatment groups (*x* axis)

comparison, the median time for tumours to reach 200 $mm^2$ in the $E_2$-supplemented group (experiment 1) was just 14 days. This was significantly faster than for 0.9% NaCl and placebo control groups in the same experiment (31.5 days, $P = 0.004$ and 42 days, $P = 0.006$ respectively).

Data for all animals in the four treatment groups common to the three experiments (anti-GnRH Ab, 0.9% NaCl, tamoxifen, placebo) were combined. The proportion of animals with tumours smaller than 200 $mm^2$ was plotted against time (Figure 4) and the median times to this event are shown (Table 1, column 4). The median times were 71.6 days for anti-GnRH Ab, 29.4 days for 0.9% NaCl, 59.4 days for tamoxifen and 37.7 days for placebo. The median time taken for tumours to reach 200 $mm^2$ was significantly longer with anti-GnRH Ab treatment than for 0.9% NaCl controls ($P = 0.0002$). Tamoxifen treatment compared with placebo controls was not significant, though the median time with tamoxifen treatment was significantly longer than that for 0.9% NaCl controls ($P = 0.037$). There was no significant difference between anti-GnRH Ab treatment and tamoxifen.

### ER and PR in tumours

Nuclear staining of tumour cells for ER or PR was noted similar to that seen in human primary tumours. Epithelial tumour cells were scored for ER or PR staining and there was no staining with a negative control antibody. Mean H-scores for ER and PR expression in tumours from the three experiments are shown in Table 2. Tumours within each treatment group appeared similar, but there were differences in the levels of receptor expression between different treatment groups.

ER expression was evident in most tumours in keeping with the fact that MCF7 is known to express ER. H-scores were higher in anti-GnRH Ab-treated tumours than 0.9% NaCl controls ($P = 0.007$). ER expression was absent in the tumours of mice that received $E_2$ supplementation. In the first experiment, tumours treated with tamoxifen did not express ER. In two subsequent experiments, there was a non-significant trend ($P = 0.07$) towards lower ER expression in tumours treated with tamoxifen compared with controls.

PR expression was highest in tumours where $E_2$ supplementation had continued, with 70% of cells exhibiting moderate to intense staining. In tumours from mice not receiving $E_2$ supplementation, H-scores were much lower for PR expression (than for ER) with very few tumour cells staining with variable intensity. In the first experiment, PR were expressed by most tumours, but levels were reduced by anti-GnRH Ab treatment. In the other two experiments, PR levels were very low in both anti-GnRH Ab and control groups. There was a trend towards elevation of PR levels in tumours treated with tamoxifen ($P = 0.08$).

There was correlation of serum $E_2$ concentrations with PR levels, and inverse correlation of ER with PR (Spearman's ρ rank correlation coefficients of 0.791 and –0.813 respectively, both $P < 0.001$).

### Effect of treatment on the reproductive organs

### *Organ weights*

Weights of the reproductive organs (ovaries and uteri) were in a similar range for 0.9% NaCl, placebo and IgG control animals (data not shown). For this reason these data were combined (median 90 mg, IQ range 70–138, $n = 29$). In mice treated with anti-GnRH Ab ($n = 18$), the reproductive organs weighed significantly less than those in controls (median 40 mg, IQ range 33–50 mg, Mann–Whitney $P < 0.001$). Organs from tamoxifen-treated animals ($n = 10$) were significantly heavier than those from controls (median 119 mg, IQ range 105–212 mg, Mann–Whitney $P = 0.034$).

### *Histology*

Examination of H&E-stained sections (Figure 5) of paraffin-fixed material from animals in the control groups revealed normal

© Cancer Research Campaign 1999

follicular development and luteinization in ovaries together with normal endometrial development of glandular structures and active stroma in uteri. Ovaries and uteri from the anti-GnRH Ab-treated mice were approximately half the size of those in controls and exhibited reduced follicular development and non-luteinized stroma in the ovaries and atrophy of the uteri with fewer glandular components and inactive stroma. In tamoxifen-treated animals, ovaries showed follicular development with minimal/variable luteinization and gross benign cystic hyperplasia of the endometrium.

### *Serum $E_2$*

The concentration of $E_2$ in serum from treated and control animals was measured by RIA (Figure 6). Basal $E_2$ levels of 23.7 ± 1.8 and 23.0 ± 6.7 pM per litre were recorded for 0.9% NaCl and placebo controls respectively. Serum $E_2$ levels were in excess of 900 pM per litre in $E_2$-supplemented animals, but fell to basal levels 1 week following removal of the pellet (data not shown). $E_2$ serum levels were significantly reduced by anti-GnRH Ab treatment (5.9 ± 1.7 pM per litre) in comparison with both 0.9% NaCl and placebo control groups (both $P = 0.04$). Rabbit IgG reduced serum $E_2$ to a level intermediate between anti-GnRH Ab treatment and 0.9% NaCl controls in the one experiment where it was included as a control (8.5 pM per litre). $E_2$ serum levels in tamoxifen-treated animals (17.0 ± 5.5 pM per litre) were not significantly different from those in controls.

### *Serum LH*

Serum LH measurements were within a range of 0.2–3.0 ng $ml^{-1}$ and are expressed as a percentage of LH measured in the serum of 0.9% NaCl-treated control animals (Figure 7.). LH was significantly reduced to 53 ± 14% ($P = 0.04$) in anti-GnRH Ab-treated animals compared with controls. Serum LH levels were similar in tamoxifen-treated animals and controls.

### *Toxicity*

Treatments were well-tolerated without signs of toxicity. Weight loss was more pronounced in some mice after prolonged treatment with tamoxifen, in 3/29 cases necessitating termination before tumours had attained the target size. However, there was no significant difference in the overall weights of mice in different treatment groups.

### DISCUSSION

The growth of MCF7 human breast tumour xenografts in nude mice was significantly inhibited by treatment with anti-GnRH antibodies compared to controls. Tamoxifen also significantly inhibited tumour growth compared to 0.9% NaCl controls (Figure 4) but was not statistically different from placebo controls within the time scale of these experiments. These results parallel work by other groups using the nude mouse model who have demonstrated tamoxifen to be tumoristatic rather than tumoricidal on the growth of MCF7 xenografts (Osborne et al, 1985; Gottardis et al, 1988). In animals treated with anti-GnRH antibodies, the time taken for tumours to reach a target size (200 $mm^2$) was longer than in those given tamoxifen (71 vs 59 days respectively). Whilst there was no significant difference between these two groups, the result shows that anti-GnRH Ab were at least as effective as the anti-oestrogen, tamoxifen in slowing the growth of established tumours at the dosages used in this study.

Agonists and antagonists of luteinizing hormone releasing hormone (LHRH) have been shown to be inhibitory to the growth of MCF7-MIII xenografts, and to reduce serum levels of both $E_2$ and LH in treated animals (Yano et al, 1992). Passive infusion of anti-GnRH Ab had a similar effect resulting in suppression of serum $E_2$ to very low levels, and also reducing serum LH levels compared to those in controls. The GnRH agonist, goserelin, is known to reduce follicle-stimulating hormone (FSH) and LH levels in premenopausal patients with breast cancer, with consequent reduction of $E_2$ and progesterone to castrate levels (Williams et al, 1986; Blamey et al, 1992). In the present study, passive immunization of mice with anti-GnRH Ab appeared to cause similar suppression of the pituitary ovarian axis.

Further evidence for suppression of pituitary gonadal function by anti-GnRH antibodies was the reduced ovary/uterine weights and the histological findings indicating lack of follicular development, no luteinization of the ovaries and inactive endometrium. The ovarian changes reported in this study, in anti-GnRH Ab-treated mice, are similar to those previously reported in breast cancer patients treated with the GnRH analogue, goserelin (Zoladex) (Williamson et al, 1988), their two major observations being lack of corporus luteum and increased frequency of cysts indicative of atretic Graafian follicles.

Chemical oophorectomy (e.g. goserelin) in premenopausal patients reduces the level of circulating $E_2$ compared to that found in post-menopausal patients and is able to induce therapeutic remissions (Williams et al, 1986; Blamey et al, 1992). MCF7 tumour xenografts treated with anti-GnRH Ab appeared to respond to reduced $E_2$ availability by up-regulation of ER and down-regulation of PR. These findings are in keeping with previous literature on the effect of $E_2$ on steroid hormone receptors in normal tissues and in MCF7 breast cancer cells (Kraus and Katzenellenbogen, 1993; Cho et al, 1994). Anti-GnRH Ab were not directly inhibitory to MCF7 tumour cell proliferation in vitro (data not shown), supporting an indirect mechanism in vivo as the most likely explanation of the tumour inhibitory effects of anti-GnRH antibodies.

A recent report has shown anti-GnRH immunization to be effective against mammary cancer in a rat syngeneic model (Ferro and Stimpson, 1997). We have shown the strategy can also be effective against a human breast cancer.

To summarize, this study has shown that passive immunization with anti-GnRH Ab in nude mice resulted in reduced levels of circulating $E_2$. This supports evidence from studies in rabbits where active immunization with the GnRH immunogen (GnRH Pharmaccine) resulted in castrate levels of sex hormones and effective contraception. GnRH Pharmaccine appears to be a novel method of producing a castrate state. In this study, anti-GnRH Ab showed enhanced therapeutic efficacy on the growth of a breast tumour xenograft comparable with tamoxifen, which is the current anti-oestrogen of choice as first-line endocrine therapy in post-menopausal patients with breast cancer.

### ACKNOWLEDGEMENTS

The authors would like to thank Aphton Corporation for funding and provision of rabbit anti-GnRH antibodies; M Dowsett, Royal Marsden, for $E_2$ serum assay; Cancer Studies Unit, University of Nottingham, for laboratory facilities; TM Morris and M Exton for technical assistance with in vivo studies; and J Bell, Histopathology, City Hospital, for immunocytochemistry.

© Cancer Research Campaign 1999

### REFERENCES

Beatson GT (1896) On the treatment of inoperable cases of carcinoma of the mamma: suggestions for a new method of treatment with illustrative cases. *Lancet* **ii**: 104–107

Blamey RW, Jonat W, Kaufmann M, Raffaele Bianco A and Namer M (1992) Goserelin depot in the treatment of premenopausal advanced breast cancer. *Eur J Cancer* **28A**: 810–814

Cho H, Aronica SM and Katzenellenbogen BS (1994) Regulation of progesterone receptor gene expression in MCF7 breast cancer cells: a comparison of the effects of cyclic adenosine 3′,5′-monophosphate, oestradiol, insulin-like growth factor-1 and serum factors. *Endocrinology* **134**: 658–664

Dowsett M, Goss PE, Powles TJ, Hutchinson G, Brodie AMH, Jeffcoate SL and Coombes RC (1987) Use of the aromatase inhibitor 4 hydroxyandrostenedione in postmenopausal breast care: optimisation of therapeutic dose and route. *Cancer Res* **47**: 1957–1961

Ferro VA and Stimpson WH (1997) Immunoneutralisation of gonadotrophin releasing hormone: a potential treatment for oestrogen-dependent breast cancer. *Eur J Cancer* **33**: 1468–1478

Gottardis MM, Robinson SP and Jordan VC (1988) Oestradiol-stimulated growth of MCF7 tumours implanted in athymic mice: a model to study the tumouristatic action of tamoxifen. *J Steroid Biochem* **30**: 311–314

Goulding H, Pinder S, Cannon P, Pearson D, Nicholson R, Snead D, Bell J, Elston CW, Robertson JF, Blamey RW and Ellis IO (1995) A new immunohistochemical antibody for the assessment of oestrogen receptor status on routine formalin fixed tissue samples. *Hum Pathol* **26**: 291–294

Jacobs E, Watson SA, Hardcastle JD and Robertson JFR (1996) Oestrogen and progesterone receptors in gastrointestinal cancer cell lines. *Eur J Cancer* **32A**: 2348–2353

Kraus WL and Katzenellenbogen BS (1993) Regulation of progesterone receptor gene expression and growth in the rat uterus: modulation of oestrogen actions by progesterone and sex steroid hormone antagonists. *Endocrinology* **132**: 2371–2379

Lee ET and Desu MM (1972) A computer programme for comparing k samples with right censored data. *Comp Progr Biomed* **2**: 315–321

Lett H (1905) An analysis of ninety nine cases of inoperable carcinoma of the breast treated by oophorectomy. *Lancet* **i**: 227–228

Osborne KC, Hobbs K and Clark GM (1985) Effect of oestrogens and anti-oestrogens on growth of human breast cancer cells in athymic nude mice. *Cancer Res* **45**: 584–590

Williams MR, Walker ILJ, Turkes A, Blamey RW and Nicholson RI (1986) The use of LH-RH agonist (ICI 118,630, Zoladex) in advanced premenopausal breast cancer. *Br J Cancer* **53**: 629–636

Williamson K, Robertson JFR, Ellis IO, Elston CW, Nicholson RI and Blamey RW (1988) Effect of LHRH agonist, Zoladex® on ovarian histology. *Br J Surg* **75**: 595–596

Yano T, Korkut E, Pinski J, Szepeshazi K, Milovanovic S, Groot K, Clarke R, Comaru-Schally AM and Schally AV (1992) Inhibition of growth of MCF-7 MIII human breast carcinoma in nude mice by treatment with agonists or antagonists of LH-RH. *Br Cancer Res Treat* **21**: 35–45

*© Cancer Research Campaign 1999*
